# Prevalence and Clinical Correlates of Autistic Traits Across Eating Disorder Diagnoses: A Retrospective Analysis of Clinical Service Data

**DOI:** 10.3390/nu18172906

**Published:** 2026-09-04

**Authors:** Adia Meyer, Lauren Makin, Karina Allen, Kate Tchanturia

**Affiliations:** 1 Department of Eating Disorders, South London and Maudsley NHS Foundation Trust, London SE5 8AZ, UK; adia.meyer@kcl.ac.uk (A.M.); karina.allen@kcl.ac.uk (K.A.); 2 Department of Psychological Medicine, Institute of Psychiatry, Psychology & Neuroscience, King’s College London, London SE5 8AF, UK; lauren.makin@kcl.ac.uk; 3 National Institute for Health and Care Research (NIHR) Maudsley Biomedical Research Centre at South London and Maudsley NHS Foundation Trust and King’s College London, London SE5 8AF, UK; 4 Department of Culture and Communication, Aalborg University, 9000 Aalborg, Denmark

**Keywords:** anorexia nervosa (AN), bulimia nervosa (BN), autistic traits, functional difficulties, avoidant/restrictive food-intake disorder (ARFID)

## Abstract

**Background**: Autistic traits are overrepresented among individuals with anorexia nervosa (AN) and are associated with greater eating disorder (ED) psychopathology, psychological distress, and work and social functioning difficulties in this population. However, less is known about the prevalence and clinical associations of autistic traits in other ED presentations, such as bulimia nervosa (BN). This study examined the prevalence of autistic traits and their associations with ED psychopathology, psychological distress, and work and social functioning difficulties in a transdiagnostic ED sample. **Methods**: A retrospective cross-sectional analysis was conducted using routinely collected clinical data of adults attending a specialist ED service with restrictive AN (AN-R), binge–purge AN (AN-BP), BN, binge eating disorder (BED), atypical AN (A-AN), or avoidant/restrictive food-intake disorder (ARFID). Prevalence rates of elevated autistic traits (AQ-10 ≥6) were described across diagnoses. Multiple linear-regression analyses examined associations between autistic traits and measures of eating disorder psychopathology, psychological distress, and work and social functioning difficulties. **Results**: Elevated autistic traits were reported by 40.0% of patients with ARFID, 34.2% with AN-BP, 31.9% with AN-R, 27.0% with BN, 26.4% with A-AN, and 16.7% with BED. Higher AQ-10 scores were significantly associated with greater ED psychopathology, psychological distress, and work and social functioning difficulties across the AN-R, BN, and full samples. In the AN-R and full samples, the associations remained significant across all models after controlling for age, gender, and ED duration. In the BN sample, all associations remained significant following adjustment except for ED psychopathology, which was no longer significant after controlling for ED duration. **Conclusions**: Autistic traits are elevated across multiple ED diagnoses and are associated with greater clinical severity. The findings support the clinical relevance of autistic traits across ED presentations and support the need for further investigation of autism-informed approaches across transdiagnostic ED services.

## 1. Introduction

Autistic traits, as measured by variations of the Autism Quotient [1,2], have been increasingly recognised as relevant to the clinical presentation of eating disorders (EDs). A growing body of research suggests that individuals accessing ED services report elevated levels of autistic traits compared to community non-ED comparison samples [3,4].

### 1.1. Prevalence and Clinical Correlates of Autistic Traits in AN

The association between autistic traits and anorexia nervosa (AN) is particularly well established. A meta-analysis of 22 studies found significantly elevated autistic traits among individuals with AN compared to healthy controls [4]. Similarly, an earlier systematic review reported that approximately 35% of individuals with AN scored above the clinical cut-off for autistic traits [5], further highlighting the high prevalence of clinically significant autistic traits in this population.

Beyond prevalence, autistic traits have been associated with increased psychosocial difficulties in AN. Higher levels of autistic traits have been linked to greater ED psychopathology [4,6], psychological distress [6,7], and work and social functioning difficulties [6,8]. For example, Tchanturia and colleagues [6] found that autistic traits were positively associated with ED psychopathology, symptoms of depression and anxiety, and work and social functioning difficulties among individuals receiving inpatient treatment for AN. Although Li and colleagues [9] replicated these findings in inpatient settings, associations were not consistently observed in day-patient or outpatient samples. Higher autistic traits have also been associated with elevated alexithymia, greater social anhedonia, and increased social, emotional, and peer difficulties [8,10]. Furthermore, individuals with higher autistic traits appear more likely to require intensive treatment, including specialist inpatient care, psychiatric admissions, and day-patient programmes, and to experience longer treatment episodes and greater cumulative time spent in hospital [7,8,11,12]. Collectively, these findings suggest that elevated autistic traits may be associated with greater clinical complexity and support needs among individuals with AN.

However, previous studies have rarely examined whether these associations remain significant after accounting for potentially important demographic and illness-related factors, such as age, gender, and ED duration [6]. Including such factors is important as some debate remains regarding the nature of autistic traits observed in AN. While some authors have proposed that AN may represent a female presentation of autism, and thus the traits are a result of autism itself [13], others have questioned whether elevated autistic traits partly reflect state-related effects associated with illness duration or starvation [14,15,16]. A recent scoping review found longitudinal and qualitative evidence reporting autistic traits in childhood, a lack of association between BMI and autistic traits, and a link between autistic traits and EDs not typically associated with low body weight. Thus, the authors posited that the association between autistic traits and EDs is not solely due to acute illness effects [17]. Nevertheless, controlling for gender and ED duration is important for establishing the nature and strength of any associations between autistic traits and EDs.

### 1.2. Prevalence and Clinical Correlates of Autistic Traits in Non-AN EDs

Although autistic traits have been extensively investigated in AN, considerably less research has examined their prevalence and clinical correlates in other ED diagnoses, including bulimia nervosa (BN), atypical AN (A-AN), binge eating disorder (BED), and avoidant/restrictive food-intake disorder (ARFID) [3]. Nevertheless, emerging evidence suggests that elevated autistic traits are also present across these diagnoses. For example, previous studies have found elevated autistic traits in almost half of individuals with ARFID [18]. Preliminary evidence also suggests that autistic traits may be elevated in BN and BED, although the available literature is mixed and based largely on small samples or conference abstracts [19,20,21,22,23]. Two conference abstracts reported significantly higher autistic trait scores among individuals with BN and BED than healthy controls [20,21], whereas another study found no significant difference between individuals with BN and healthy controls [22]. Two studies reported low rates of elevated autistic traits across BN, but one of them reported low rates in BED [23], whereas the other reported high rates in BED [19]. Overall, the limited evidence base and small sample sizes make it difficult to draw firm conclusions regarding the prevalence of autistic traits across non-AN ED diagnoses.

Despite growing recognition that autistic traits may be relevant across ED presentations, research examining their association with ED-related outcomes beyond AN remains limited. To date, few studies have investigated whether autistic traits are associated with ED psychopathology, psychological distress, or work and social functioning difficulties across diverse ED diagnoses.

### 1.3. Current Study

Thus, the present study aimed to provide a preliminary, hypothesis-driven examination of the prevalence and clinical correlates of autistic traits across a transdiagnostic adult ED sample.

The specific research questions were as follows:

1. What proportion of individuals in each diagnostic group (AN restrictive subtype, AN binge–purge subtype, BN, A-AN, BED, and ARFID) display elevated autistic traits (AQ-10 ≥ 6)?
2. Are autistic traits (AQ-10) associated with ED psychopathology (EDE-Q), psychological distress (CORE-10), and work and social functioning difficulties (WSAS), even when controlling for age, gender, and ED duration?

We hypothesised that higher AQ-10 scores would be significantly associated with greater ED psychopathology, psychological distress, and work and social functioning difficulties, and that these associations would remain significant after controlling for age, gender, and ED duration.

## 2. Materials and Methods

### 2.1. Data Source

This cross-sectional study used routinely collected clinical data from the specialist adult ED services in South London and Maudsley (SLaM) NHS Foundation Trust, from December 2022 to August 2025. Data was collected via self-reported questionnaires completed at assessment, the start of treatment, or at treatment end using MS Forms (See Supplementary Materials S2). Patients were informed that their data would be used for audit purposes and clinical service evaluation and had the option to opt-out of completing the measures. Patients who completed the self-report measures were included regardless of their assessment outcome or whether they were accepted for treatment in ED services. Clinical diagnoses were established following a semi-structured clinical assessment and discussion with the service multidisciplinary team and were manually recorded in the clinical database by the assessing clinician. Diagnoses were made based on the criteria outlined in the Diagnostic and Statistical Manual of Mental Disorders (5th ed.; DSM-5) and all patients included in this study had a diagnosis of AN restrictive subtype (AN-R), AN binge–purge subtype (AN-BP), BN, A-AN, BED, or ARFID [24]. Study permission was obtained from the Clinical Governance Committee Research and Development Office in 2004. No changes were made to patients’ care and no new data was collected over the duration of this study. Therefore, no additional ethical approvals were needed, and informed consent was not required in accordance with national regulations and institutional policies.

### 2.2. Measures

#### 2.2.1. Autistic Traits (AQ-10)

Patients completed the Autism Spectrum Quotient, short version (AQ-10), as part of the standard questionnaire pack [2]. The AQ-10 is a brief 10-item, self-report questionnaire, designed to screen for autism. Each item is scored as 0 or 1, with one point assigned for responses indicative of autistic traits. Total scores therefore range from 0 to 10, with higher scores indicating greater levels of autistic traits. A clinical cut-off of six or more indicates a positive screening result or elevated autistic traits but does not establish autism. The brief questionnaire has equivalent validity to the longer version [25] and studies within ED populations provide some support for its clinical utility. For example, in an outpatient ARFID service, 80.2% of individuals with an existing autism diagnosis scored above the clinical threshold, compared with 6.5% of non-autistic individuals, suggesting that the AQ-10 can discriminate between autistic and non-autistic patients in this context [18]. However, sensitivity and specificity rates were not calculated for the AQ-10 in adults due to small sample size [18]. Similarly, in a restrictive ED sample, the AQ-10 demonstrated 85% classification accuracy for distinguishing individuals with and without an autism diagnosis, although sensitivity was limited (69%), indicating potential false negative [26]. Despite these mixed findings, the AQ-10 remains the only autism screener currently recommended by NICE for adult clinical use and is one of the measures endorsed by the ED Clinical Research Network (EDCRN) [27]. In this study, Cronbach’s alpha coefficient was acceptable (*a* = 0.77).

#### 2.2.2. ED Psychopathology (EDE-Q)

The ED Examination Questionnaire (EDE-Q) was also part of the questionnaire pack. The EDE-Q is a 36-item self-report questionnaire that measures ED symptomology and behaviours across the last 28 days [28]. The questionnaire includes four subscales, dietary restraint, weight concern, shape concern, and eating concern, and a global score, which reflects overall ED psychopathology. The EDE-Q is routinely administered at admission and discharge in most UK specialist ED services [27]. In this study, Cronbach’s alpha coefficient was good (*a* = 0.92).

#### 2.2.3. Psychological Distress (CORE-10)

Clinical Outcomes in Routine Evaluation (CORE-10) is a 10-item self-report measure used to examine symptoms of psychological distress over the past week [29]. Scores can range from 0 to 40, with a clinical cut-off of 11. Items explore distress regarding mood, trauma, risk to oneself, physical problems, and general functioning. The CORE-10 has previously been used in autistic–ED populations [30]. In this study, Cronbach’s alpha coefficient was acceptable (*a* = 0.71).

#### 2.2.4. Work and Social Functioning (WSAS)

The Work and Social Adjustment Scale (WSAS) is a 5-item self-report measure that assesses an individual’s difficulties in the following domains: home management, ability to work, both private and social leisure, and their ability to form and maintain close relationships [31]. The domains are scored on a 9-point Likert scale, ranging from 0 (no difficulties) to 8 (very severe difficulties). The maximum total score for this scale is 40, with a clinical cut-off of 20 and above. In this study, Cronbach’s alpha coefficient was good (*a* = 0.85).

#### 2.2.5. Age, Gender, and ED Duration

Patients were asked their age, their gender (with the options female, male, non-binary, or other), and for how many years they had had an ED, alongside other demographic information (see Supplementary Materials S2 for full questionnaire).

### 2.3. Data Analysis

Data was manually screened and cleaned in Microsoft Excel, with duplicate cases identified and removed automatically. Patient records were identified using their unique patient code. Where multiple records were present for the same patient, duplicate records were removed so that each patient contributed only the first completed set of outcome measures to the analytic dataset. All statistical analyses were conducted using IBM SPSS Statistics for Mac (Version 32).

**Figure 1:**
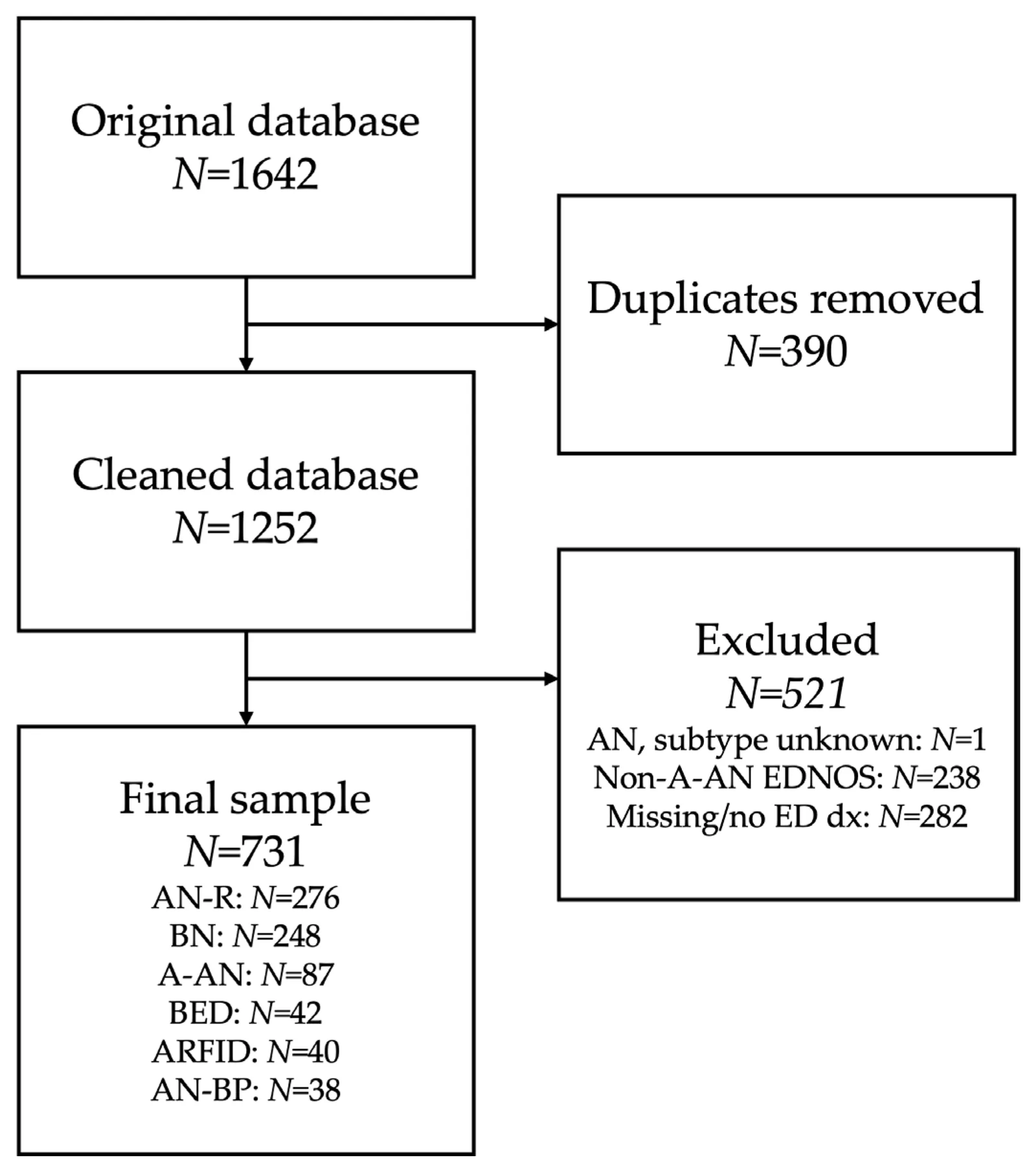
Case inclusion flowchart.

From the initial 1642 cases, duplicates were first removed leaving a remaining 1252 cases. Those without an ED diagnosis (*n* = 282), those with EDNOS other than A-AN (as this category comprised clinically heterogenous presentations; *n* = 238), and those with AN with unknown subtype (*n* = 1) were excluded prior to analysis, leaving a final study sample of 731 cases (see Figure 1).

No cases had missing data for the outcome measures (AQ-10, EDE-Q, CORE10, or WSAS). Cases missing data on age, gender, or ED duration were retained where possible but were excluded from regression analyses requiring these variables. Missing data for variables included in the regression models was below 5% across the AN-R, BN, and full-sample analyses (see Table S1). Regression analyses were therefore conducted using complete-case deletion.

As this study used an existing clinical dataset, the sample size was determined by the number of eligible patients available rather than by an *a priori* power calculation. The smaller diagnostic subgroups were retained to provide a transdiagnostic description of autistic traits, although estimates for these groups are expected to be less precise.

To address the first research question, the proportion of patients scoring above the clinical cut-off for elevated autistic traits (AQ-10 ≥ 6) was calculated for each diagnostic group (AN-R, AN-BP, BN, A-AN, BED, and ARFID).

To address the second research question, multiple linear-regression analyses were conducted to examine associations between autistic traits and clinical outcomes while controlling for demographic and illness-related variables. Regressions analyses were only run for the AN-R sample, BN sample, and full sample, as the other diagnostic groups did not have sufficient sample sizes to be analysed independently. AQ-10 scores were the primary independent variable. For the AN-R and BN samples, covariates were entered sequentially, with age entered in Model 2, gender additionally entered in Model 3, and ED duration additionally entered in Model 4. Gender was coded as a binary variable, with female participants as the reference category and male participants as the comparison category. Non-binary/other responses (*n* = 17) were excluded from regression analyses because of the small number of participants in these categories. For the full sample, diagnosis was additionally entered as a categorical variable in Model 5, following adjustment for age, gender, and ED duration with AN-R used as the reference category. Separate analyses were conducted for the AN-R sample, the BN sample, the full sample, and for the three dependent variables: EDE-Q, CORE10, and WSAS scores.

The nine fully adjusted models, comprising three outcomes across the AN-R, BN, and full samples, were defined as the primary hypothesis-driven analyses. Sequential models examining the effects of covariate adjustment and sensitivity analyses were considered secondary. To account for multiple comparisons, Holm–Bonferroni corrections were applied across these nine primary tests, controlling the family-wise error rate at α = 0.05. Statistical significance was therefore assessed both using the unadjusted bootstrap *p* value and according to whether the result remained significant following Holm–Bonferroni correction.

Regression assumptions were assessed using examination of residual diagnostics, including linearity, homoscedasticity, normality of residuals, multicollinearity, and influential observations. Multicollinearity was assessed using tolerance and variance inflation factor (VIF) statistics (see Supplementary Materials S1, Table S13). Some deviation from normality was observed in the residuals; however, there was no evidence of highly influential observations. Linear regression was therefore retained, with 1000 bootstrap samples used to provide more robust inference. Bias-corrected and accelerated (BCa) 95% confidence intervals and two-tailed bootstrap *p* values were estimated for regression coefficients. Unstandardised regression coefficients (*B*), BCa 95% confidence intervals, adjusted *R*^2^, and bootstrap *p* values were reported. An additional sensitivity analysis was conducted, replicating the methods of Tchanturia et al. [6] (full method and results are reported in Supplementary Materials S2).

For categorical variables, effect sizes were calculated using Cramer’s V and interpreted as small (≈0.10), medium (≈0.30), and large (≈0.50) [32].

## 3. Results

### 3.1. Sample Characteristics

A total of 731 patients were included in the analyses. The sample comprised 37.8% patients with AN-R (*n* = 276), 33.9% with BN (*n* = 248), 11.9% with A-AN (*n* = 87), 6% with BED (*n* = 42), 5.8% with ARFID (*n* = 40), and 5.2% with AN-BP (*n* = 38). Mean ED duration was 11.08 years (SD = 9.9). Most patients were treated in an outpatient setting (91%), with the remainder receiving day-patient, enhanced treatment team, or inpatient care. Questionnaire completion occurred predominantly at assessment or treatment start (93.8%, *n* = 686), with only 4.1% (*n* = 30) of questionnaires completed at treatment end. A further 2.1% (*n* = 15) were either missing timing information or completed at other time points.

The mean age was 28.83 (SD = 9.7; range = 17 to 64). One patient aged 17 years was included in the adult sample because they were transferring from the adolescent service to adult ED services and had completed the intake measures in preparation for this transfer. Most patients identified as female (86.9%), heterosexual (65.8%), and White (69.8%). The highest level of education most frequently reported was a university degree (32.1%), followed by an upper-secondary or vocational qualification (A level/NVQ; 21.3%), postgraduate degree (18.3%), other secondary vocational diplomas (BTEC; 10.7%), lower-secondary qualification (O level/GCSE; 11.1%), and no formal qualifications (2.1%). Demographic differences between diagnostic groups were small to moderate (Cramer’s *V* = 0.08–0.23; see Supplementary Materials S1, Table S2).

### 3.2. Prevalence of Elevated Autistic Traits

**Figure 2:**
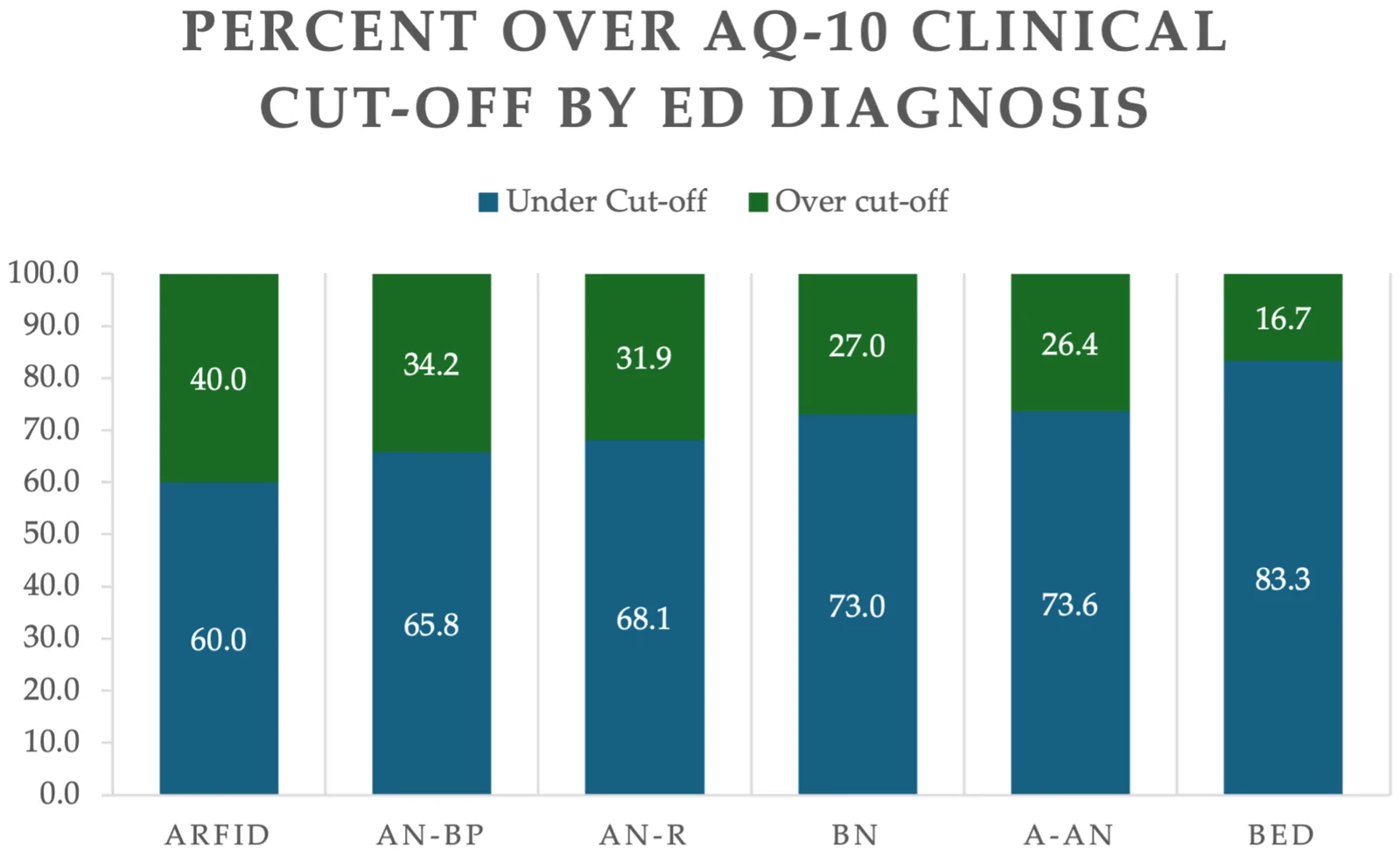
Proportion of participants scoring above the AQ-10 clinical cut-off by ED diagnosis. Note: The proportion of patients scoring above the clinical cut-off for elevated autistic traits (AQ-10 ≥ 6) was 31.9% (*n* = 276; 95% CI 26.4–37.7%) in the AN-R group, 34.2% (*n* = 38; 95% CI 19.6–51.4%) in the AN-BP group, 27.0% (*n* = 248; 95% CI 21.6–33.0%) in the BN group, 26.4% (*n* = 87; 95% CI 17.6–37.0%) in the A-AN group, 16.7% (*n* = 42; 95% CI 7.0–31.4%) in the BED group, and 40.0% (*n* = 40; 95% CI 24.9–56.7%) in the ARFID group.

All patients completed the AQ-10 (*n* = 731). The mean was 4.37 (SD = 2.48) with a range of 10; 29.3% (*n* = 214) of patients scored above the clinical cut-off. The proportion of patients scoring above the AQ-10 clinical cut-off (≥6) varied across diagnostic groups (Figure 2). Elevated autistic traits were reported by 40.0% of individuals with ARFID, 34.2% with AN-BP, 31.9% with AN-R, 27.0% with BN, 26.4% with A-AN, and 16.7% with BED.

### 3.3. Associations Between Autistic Traits and Other Measures

Multiple linear-regression analyses were conducted to examine associations between autistic traits (AQ-10 scores) and ED psychopathology (EDE-Q Global), psychological distress (CORE-10), and work and social functioning difficulties (WSAS) in the AN-R, BN, and full samples. Analyses were conducted using complete cases. For the AN-R and BN samples, fully adjusted models included age, gender, and ED duration. For the full sample, the models were additionally adjusted for diagnostic group, entered as a categorical variable. The nine fully adjusted models, comprising three outcomes across three samples, were considered the primary analyses. Regression coefficients were estimated using 1000 bootstrap samples, with bias-corrected and accelerated (BCa) 95% confidence intervals. Holm–Bonferroni correction was applied across the nine primary tests.

Higher AQ-10 scores were associated with higher EDE-Q Global scores in the AN-R and full samples, but not in the BN sample after full adjustment. In the AN-R sample, each one-point increase in the AQ-10 score was associated with a 0.122-point increase in the EDE-Q Global score (95% BCa CI 0.045–0.205, *p* < 0.001, Holm–Bonferroni significant). In the BN sample, the corresponding association was smaller and significant only when adjusting for age and gender. The BN group did not reach statistical significance when adjusting for ED duration (B = 0.055, 95% BCa CI −0.007–0.115, *p* = 0.074). In the full sample, each one-point increase in the AQ-10 score was associated with a 0.097-point increase in the EDE-Q Global score (95% BCa CI 0.057–0.137, *p* < 0.001, Holm–Bonferroni significant).

For psychological distress, higher AQ-10 scores were significantly associated with higher CORE-10 scores across all three samples. In the AN-R sample, each one-point increase in the AQ-10 score was associated with a 0.783-point increase in the CORE-10 score (95% BCa CI 0.487–1.070, *p* < 0.001, Holm–Bonferroni significant). Corresponding associations were observed in the BN sample (B = 0.683, 95% BCa CI 0.394–0.975, *p* < 0.001, Holm–Bonferroni significant) and full sample (B = 0.744, 95% BCa CI 0.590–0.900, *p* < 0.001, Holm–Bonferroni significant).

Similarly, higher AQ-10 scores were significantly associated with greater work and social functioning difficulties across all three samples. In the AN-R sample, each one-point increase in the AQ-10 score was associated with a 1.117-point increase in the WSAS score (95% BCa CI 0.700–1.502, *p* < 0.001, Holm–Bonferroni significant). Associations were also observed in the BN sample (B = 1.118, 95% BCa CI 0.666–1.645, *p* < 0.001, Holm–Bonferroni significant) and full sample (B = 1.223, 95% BCa CI 0.952–1.470, *p* < 0.001, Holm–Bonferroni significant).

Adjusted R^2^ values for the fully adjusted models ranged from 0.033 to 0.234 (Table 1). Detailed hierarchical regression results for Models 1–4 in the AN-R and BN samples and Models 1–5 in the full sample, including changes in R^2^ and multicollinearity statistics, are presented in Supplementary Tables S4–S13.

Across the sequential regression models, the associations between AQ-10 scores and CORE-10 and WSAS scores were generally maintained following adjustment for age, gender, and ED duration. For EDE-Q Global, associations remained significant following adjustment in the AN-R and full samples but were not statistically significant in the BN sample following adjustment for ED duration. In the full-sample analyses, additionally adjusting for eating-disorder diagnosis substantially increased the variance explained in EDE-Q scores (ΔR^2^ = 0.206), with smaller increases for CORE-10 (ΔR^2^ = 0.022) and WSAS (ΔR^2^ = 0.021). The AQ-10 coefficient increased slightly following adjustment for diagnosis for EDE-Q (B = 0.097, 95% BCa CI [0.057, 0.137]), CORE-10 (B = 0.744, 95% BCa CI [0.590, 0.900]), and WSAS (B = 1.223, 95% BCa CI [0.952, 1.470]); all *p* < 0.001. Full regression results for each sequential model are presented in Supplementary Tables S4–S12.

Kendall’s tau-b correlations (Table S14) produced findings consistent with the primary analyses.

## 4. Discussion

This cross-sectional study examined the prevalence of elevated autistic traits and their associations with self-report measures across ED diagnoses in a sample of adult patients at an ED specialist service. Elevated autistic traits were reported by 40.0% of individuals with ARFID, 34.2% with AN-BP, 31.9% with AN-R, 27.0% with BN, 26.4% with A-AN, and 16.7% with BED. Higher autistic traits were associated with greater ED psychopathology, psychological distress, and work and social functioning difficulties across the AN-R, BN, and the full samples. These associations remained significant after adjustment for age and gender, and additionally for ED duration in the AN-R and full samples. In BN, associations with psychological distress and work and social functioning difficulties remained significant after adjustment for ED duration, whereas the association with ED psychopathology did not.

### 4.1. Prevalence of Elevated Autistic Traits

The prevalence findings extend the existing literature by providing estimates across multiple ED diagnoses within a single clinical sample. Consistent with previous research, elevated autistic traits were most common in ARFID and AN, supporting evidence that autism and autistic traits are particularly associated with restrictive eating presentations [4,18,20,22]. The prevalence of elevated autistic traits in the AN-BP (34.2%; 95% CI 19.6–51.4%) and AN-R samples (31.9%; 95% CI 26.4–37.7%) was broadly consistent with previous estimates that approximately 35% of individuals with AN had elevated autistic traits [5]. Similarly, the prevalence in the ARFID group (40.0%; 95% CI 24.9–56.7%) was only slightly lower than the 47.2% previously reported [18].

In contrast, the prevalence of autistic traits observed in BN (27.0%; 95% CI 21.6–33.0%) and BED (16.7%; 95% CI 7.0–31.4%) differed considerably from previous estimates. Earlier studies have reported considerably lower rates in BN (approximately 4–6%) and highly variable estimates in BED (0–60%) [19,23]. However, these studies were based on very small samples (BN: *n* = 16–23; BED: *n* = 5–10), limiting the precision and generalisability of their findings. By comparison, the present study included substantially larger BN (*n* = 248) and BED (*n* = 42) samples, providing more precise preliminary estimates while highlighting the need for further replication across diverse clinical populations.

It is also interesting that the prevalence of elevated autistic traits was higher in AN-BP than AN-R, and in BN than in A-AN, suggesting that restriction may not be the only relevant feature of EDs for autistic traits. Furthermore, although elevated autistic traits were less common in BN, A-AN, and BED than in ARIFD or AN, substantial proportions of patients in these groups (>16%) still scored above the AQ-10 clinical cut-offs. Given the limited literature examining autistic traits across non-AN diagnoses, these findings provide useful preliminary prevalence estimates and further support the relevance of autistic traits across a broader spectrum of ED presentations and within non-underweight populations [3]. However, the relatively small sample sizes for AN-BP, ARFID, and BED warrant cautious interpretation and replication in larger samples.

### 4.2. Associations Between Autistic Traits and Self-Report Measures

Overall, the findings largely supported the hypotheses. Higher autistic traits were associated with greater ED psychopathology, psychological distress, and work and social functioning difficulties, and these associations remained significant after adjustment for age, gender, and ED duration in the AN-R and full samples. In BN, associations with psychological distress and work and social functioning difficulties remained significant after adjustment, whereas the association between autistic traits and ED psychopathology was no longer statistically significant after adjustment for ED duration.

Adjustment for eating disorder diagnosis did not substantially alter the associations between autistic traits and clinical outcomes, with associations remaining evident across EDE-Q, CORE-10, and WSAS. However, diagnosis accounted for considerable additional variation in ED psychopathology, with a more limited contribution to psychological distress and work and social functioning difficulties. These findings suggest that the observed associations between autistic traits and clinical outcomes are not attributable solely to differences in diagnostic composition.

These findings align with previous research conducted in AN samples, which demonstrated associations between autistic traits and greater ED psychopathology, psychological distress, and work and social functioning difficulties [4,6,7,8,9]. Importantly, the present study extends this literature in several ways. First, previous studies have primarily focused on inpatient AN samples, and findings outside of inpatients have been more mixed [9], whereas the current study included a broader clinical population spanning inpatient, day-patient, and outpatient settings. Second, the present study examined multiple domains of clinical functioning rather than focusing solely on ED psychopathology. Third, and most importantly, comparable associations were largely observed within BN and across the wider transdiagnostic sample.

The extension of these findings to BN represents a potentially important contribution. To our knowledge, no previous study has specifically examined the clinical correlates of autistic traits in a BN sample. Although effect sizes were generally stronger in AN-R than BN, significant associations remained evident across all three clinical measures and remained significant after adjustment for age and gender. This suggests that the clinical relevance of autistic traits may extend beyond restrictive ED presentations and may represent a broader transdiagnostic phenomenon [3,33].

Several mechanisms may contribute to the observed associations. Previous literature has highlighted potential roles for sensory processing differences, cognitive styles, social communication differences, and neuro-minority stress in shaping both ED symptoms, distress, and difficulties among autistic individuals [33,34,35,36,37,38]. However, these mechanisms were not directly examined in the present study, and future research is needed to determine the pathways through which autistic traits may contribute to increased psychopathology, distress, and work and social functioning difficulties across ED diagnoses.

The persistence of association adjustment for ED duration in the AN-R and full sample provides some evidence these relationships are not solely explained by illness chronicity [17]. However, this should not be interpreted as definitively demonstrating that illness chronicity does not contribute to these associations, particularly given that ED duration was self-reported and may have been measured imprecisely. In BN, the attenuation of the relationship between autistic traits and ED psychopathology following adjustment for ED duration may indicate that illness duration accounts for some of the observed relationship. Alternatively, ED duration may act as a marker of other unmeasured factors, such as illness severity, treatment history, or cumulative psychological distress, that contribute to both autistic traits and ED psychopathology. Given the cross-sectional design, it is not possible to determine whether ED duration is a confounding factor, a marker of other clinical characteristics, or part of the pathway linking autistic traits and ED psychopathology.

Furthermore, the presence of significant associations within BN, a diagnosis not typically characterised by prolonged starvation or very low BMI, may suggest that the association between autistic traits and ED psychopathology extends beyond the effects of malnutrition alone. However, current BMI and nutritional status were not included in the analyses, and the potential contribution of nutritional status therefore remains unclear.

### 4.3. Clinical Implications

The present findings suggest that individuals with elevated autistic traits may present to ED services with greater clinical complexity, including increased ED psychopathology, psychological distress, and work and social functioning difficulties. These findings may help explain previous reports that individuals with higher autistic traits appear more likely to require intensive treatment, including specialist inpatient care, psychiatric admissions, and day-patient programmes, and to experience longer treatment episodes and greater cumulative time spent in hospital [7,8,11,12].

Although the present study did not evaluate treatment adaptations or clinical outcomes, the findings highlight the potential importance of recognising autistic traits within ED services. Early identification may help clinicians consider whether additional assessment or individually tailored support is appropriate, particularly for patients presenting with greater psychological distress or functional impairment [30,39]. The present findings therefore provide further rationale for investigating autism-informed models of care, such as PEACE (www.peacepathway.org) [40], across a broader range of ED presentations.

Potential adaptations proposed in previous literature include clearer and more explicit communication, greater flexibility in treatment delivery, sensory accommodations, and additional support for emotional awareness and interoception [34,35,40]. While many of these approaches have primarily been developed within AN services, future research should evaluate their effectiveness across transdiagnostic ED populations, including those with BN.

### 4.4. Strengths and Limitations

A key strength of the present study is that it replicates previous research demonstrating associations between autistic traits and ED psychopathology, psychological distress, and work and social functioning difficulties while extending this across multiple ED diagnoses. In particular, the inclusion of BN, A-AN, BED, and ARFID contributes to the growing transdiagnostic literature examining neurodivergence within ED populations [30,33,35,41]. The use of multiple linear-regression analyses also enabled examination of these associations while controlling for potential demographic and illness-related confounders. A further strength is the use of routinely collected clinical data from a large specialist ED service. This allowed this study to examine autistic traits within real-world clinical settings, increasing the ecological validity and clinical relevance of the findings.

However, several limitations should also be acknowledged. The cross-sectional design prevents conclusions regarding causality or the direction of observed relationships. Longitudinal studies are needed to determine whether elevated autistic traits contribute to poorer clinical outcomes over time, or whether these associations reflect other underlying factors.

This study also relied on routinely collected clinical data, this limiting the variables available for analysis. For example, the present study was unable to utilise specific measures of anxiety and depression such as the HADS [42], instead relying on the CORE-10 as a broader measure of psychological distress. Autistic traits were measured using the AQ-10, a screening instrument rather than a diagnostic assessment. Although the AQ-10 is recommended by NICE and is widely used within ED research, it may fail to identify some autistic individuals within clinical ED samples [26]. Furthermore, formal autism diagnostic assessments were not available, meaning it was not possible to determine whether participants meeting the AQ-10 threshold would have met diagnostic criteria for autism.

Both the predictor (AQ-10) and outcome measures (EDE-Q, CORE-10, and WSAS) were self-reported. Consequently, the observed associations may have been inflated by common-method variance or shared response styles. Similarly, ED duration was self-reported and participants may have differed in how they conceptualised illness onset, although analyses adjusted for age, gender, and ED duration, residual confounding remain possible. Factors such as BMI, treatment setting, psychiatric comorbidities, medication use, illness severity, and other neurodevelopmental conditions, particularly ADHD [30], may have also influenced the observed associations, but we did not have adequate information to control for these.

Completion of the questionnaire battery was optional, introducing the possibility of selection bias if participants who completed questionnaires differed systematically from those who did not. Although we are able to compare demographics of patients who missed certain questions, we do not have information on patients who did not complete any self-report measures, preventing assessment of the extent of this bias. Additionally, while most participants (93.8%) completed questionnaires at assessment or treatment initiation, a small proportion completed measures at later stages of treatment. Because symptom severity and psychosocial functioning may change over the course of treatment, we cannot completely exclude the possibility that variation in questionnaire timing influenced the observed relationships. However, the predominance of baseline assessments suggests that any such effect is likely to be limited.

Our sample excluded individuals without a confirmed ED diagnosis. In addition, although the overall sample was large, the AN-BP, A-AN, BED, and ARFID groups were relatively small, limiting diagnosis-specific analyses and the precision of prevalence estimates. This study was conducted within a single ED service, which may limit the generalisability of the findings. Replication across multiple services and healthcare settings is therefore needed, and large-scale clinical research initiatives such as EDCRN [27] may provide opportunities to examine these relationships within larger and more representative samples in the future. Although findings were generally consistent across analyses, the relatively large number of statistical tests also increases the possibility of Type I error, and some statistically significant findings should therefore be interpreted cautiously.

## 5. Conclusions

Elevated autistic traits were observed across multiple ED diagnoses and were associated with greater concurrent ED psychopathology, psychological distress, and work and social functioning difficulties. These associations were observed not only in AN-R but also in BN and the wider transdiagnostic sample, highlighting the clinical relevance of autistic traits beyond restrictive ED presentations. Future prospective research using validated autism assessments is needed to clarify the mechanisms underlying these associations and determine the implications of autistic traits for assessment, treatment planning, and service provision across ED diagnoses.

## Figures and Tables

**Table 1:** Fully adjusted complete-case regression models.

Measure	Sample	*B*	95% BCa CI	*n*	Adjusted R^2^	Holm-Adjusted *p*
EDE-Q	AN-R	0.122	(0.045, 0.205)	(254)	0.137	<0.001 *
	BN	0.055	(−0.007, 0.115)	(232)	0.033	0.074
	Full sample	0.097	(0.057, 0.137)	(680)	0.234	<0.001 *
CORE10	AN-R	0.783	(0.487, 1.070)	(254)	0.179	<0.001 *
	BN	0.683	(0.394, 0.975)	(232)	0.062	<0.001 *
	Full sample	0.744	(0.590, 0.900)	(680)	0.119	<0.001 *
WSAS	AN-R	1.117	(0.700, 1.502)	(254)	0.192	<0.001 *
	BN	1.118	(0.666, 1.645)	(232)	0.123	<0.001 *
	Full sample	1.223	(0.952, 1.470)	(680)	0.142	<0.001 *

Note: B = unstandardised regression coefficient; BCa CI = bias-corrected and accelerated bootstrap confidence interval. *p* values are two-tailed bootstrap *p* values based on 1000 bootstrap samples. * indicates significance following Holm–Bonferroni correction across the nine primary analyses (family-wise α = 0.05). Models were adjusted for age, gender, and ED duration; full-sample models were additionally adjusted for diagnosis.

## Data Availability

The datasets presented in this article are not readily available because of National Health Services guidance. Requests to access the datasets should be directed to K.T., kate.tchanturia@kcl.ac.uk.

## Supplementary Materials

The following supporting information can be downloaded at: https://www.mdpi.com/article/10.3390/nu18172906/s1, Table S1: Missingness data for all cases; Table S2: Demographic characteristics; Table S3: Family history of autism, support needs, absence from work and school, and history of one or more suicide attempt; Table S4: Unadjusted and adjusted linear-regression models for the EDE-Q in the AN-R sample; Table S5: Unadjusted and adjusted linear-regression models for the EDE-Q in the BN sample; Table S6: Unadjusted and adjusted linear-regression models for the EDE-Q in the full sample; Table S7: Unadjusted and adjusted linear-regression models for the CORE-10 in the AN-R sample; Table S8: Unadjusted and adjusted linear-regression models for the CORE-10 in the BN sample; Table S9: Unadjusted and adjusted linear-regression models for the CORE-10 in the full sample; Table S10: Unadjusted and adjusted linear-regression models for the WSAS in the AN-R sample; Table S11: Unadjusted and adjusted linear-regression models for the WSAS in the BN sample; Table S12: Unadjusted and adjusted linear-regression models for the WSAS in the full sample; Table S13. Multicollinearity diagnostics for the final regression models; Supplementary Materials S1: Replication of Tchanturia et al. [6]; Figure S1: Visual synthesis of Kendall’s tau-B correlation analysis findings; Table S14: Correlation between AQ-10 scores and psychopathology across diagnoses; Supplementary Materials S2: Patient intake form.

## Abbreviations

The following abbreviations are used in this manuscript:

ED	Eating Disorder
AN	Anorexia Nervosa
BN	Bulimia Nervosa
AN-BP	Anorexia Nervosa—Binge–Purge Subtype
A-AN	Atypical Anorexia Nervosa
BED	Binge Eating Disorder
ARFID	Avoidant/Restrictive Food Intake Disorder
AN-R	Anorexia Nervosa—Restrictive Subtype
BMI	Body Mass Index
NHS	National Health Service
AQ-10	Autism Spectrum Quotient, short version
EDE-Q	Eating Disorder Examination Questionnaire
CORE-10	Clinical Outcomes in Routine Evaluation
HADS	Hospital Anxiety and Depression Scale
WSAS	Work and Social Adjustment Scale
CIs	Confidence Intervals
