# Supplementary material for: Prevalence and Clinical Correlates of Autistic Traits Across Eating Disorder Diagnoses: A Retrospective Analysis of Clinical Service Data
Source: Nutrients. 2026 Sep 4;18(17):2906. doi: 10.3390/nu18172906 (PMC13567302; doi:10.3390/nu18172906)
Supplement: Supplementary file 1 [file nutrients-18-02906-s001.zip › nutrients-4485311-supplementary.pdf]

Supplementary Materials S1

Table S1. Missingness data for all cases. Shown overall and separately for the six groups. Cells where >5% of data is missing are orange.

	Overall, % (n)	AN-R, % (n)	BN, % (n)	A-AN, % (n)	BED, % (n)	ARFID, % (n)	AN-BP, % (n)
**Gender**	4.20 (31)	4.70 (13)	4.00 (10)	1.10 (1)	7.10 (3)	2.50 (1)	7.90 (3)
**Sexual orientation**	3.80 (28)	4.70 (13)	4.00 (10)	1.10 (1)	7.10 (3)	2.50 (1)	0.00 (0)
**Ethnicity**	3.80 (28)	4.70 (13)	4.00 (10)	1.10 (1)	7.10 (3)	2.50 (1)	7.90 (3)
**Level of Education**	3.80 (28)	4.70 (13)	4.00 (10)	1.10 (1)	7.10 (3)	2.50 (1)	0.00
**Service type**	0.00	0.00	0.00	0.00	0.00	0.00	0.00
**Weight trend**	6.60 (48)	7.60 (21)	6.05 (15)	3.40 (3)	7.10 (3)	5.00 (2)	10.50 (4)
**Family autism**	3.80 (26)	4.70 (13)	4.00 (10)	1.10 (1)	7.10 (3)	2.50 (1)	7.90 (3)
**Verbal support**	6.80 (50)	12.70 (35)	4.00 (10)	1.10 (1)	7.10 (3)	2.50 (1)	0.00
**Visual support**	6.80 (50)	12.70 (35)	4.00 (10)	1.10 (1)	7.10 (3)	2.50 (1)	0.00
**Absence from work**	4.20 (31)	4.70 (13)	4.00 (10)	1.10 (1)	7.10 (3)	2.50 (1)	7.90 (3)
**Absence from school**	4.20 (31)	4.70 (13)	4.00 (10)	1.10 (1)	7.10 (3)	2.50 (1)	7.90 (3)
**Suicide attempt**	4.20 (31)	4.70 (13)	4.00 (10)	1.10 (1)	7.10 (3)	2.50 (1)	7.90 (3)
**Smoking**	4.90 (36)	5.80 (16)	4.00 (10)	1.10 (1)	7.10 (3)	2.50 (1)	13.20 (5)
**Alcohol**	5.30 (39)	5.80 (16)	5.20 (13)	1.10 (1)	7.70 (3)	2.50 (1)	13.20 (5)

Table S2. Demographic characteristics. Demographics, ED information (diagnosis, service type, and weight trend), and alcohol consumption for all cases, shown overall and separately for the six groups. Cells where *n*<5 are orange and where *n*=0 are red.

	Overall, % (n)	AN-R, % (n)	BN, % (n)	A-AN, % (n)	BED, % (n)	ARFID, % (n)	AN-BP, % (n)	Effect size, *v*
**Gender**[1]								0.08
Female	86.90 (635)	87.00 (240)	86.70 (215)	92.00 (80)	81.00 (34)	80.00 (32)	89.50 (34)	
Male	6.60 (48)	5.40 (15)	7.30 (18)	5.80 (5)	11.90 (5)	12.50 (5)	0.00	
Non-binary	2.30 (17)	2.90 (8)	2.50 (5)	1.10 (1)	0.00	5.00 (2)	2.60 (1)	
**Sexual orientation**[2]								0.08
Heterosexual	65.80 (481)	63.40 (175)	66.90 (166)	75.90 (66)	57.10 (24)	67.50 (27)	60.50 (23)	
Bi/pansexual	13.00 (95)	12.70 (35)	14.10 (35)	9.20 (8)	19.00 (8)	15.00 (6)	7.80 (3)	
Lesbian/gay	5.60 (41)	5.80 (16)	7.30 (18)	3.40 (3)	2.40 (1)	2.50 (1)	5.30 (2)	
Asexual	1.50 (11)	2.20 (6)	1.20 (3)	0.00	0.00	5.00 (2)	0.00	
Queer	0.60 (5)	0.70 (2)	0.80 (2)	0.00	0.00	0.00	2.60 (1)	
**Ethnicity**								0.14
White	69.80 (510)	77.90 (215)	65.30 (162)	71.30 (62)	50.00 (21)	57.50 (23)	73.70 (28)	
Mixed	7.80 (57)	6.50 (18)	8.90 (22)	11.50 (10)	14.30 (6)	5.00 (2)	7.90 (3)	
Black	8.80 (64)	2.90 (8)	10.90 (27)	9.20 (8)	23.80 (10)	22.50 (9)	5.30 (2)	
Asian	6.00 (44)	5.40 (15)	7.70 (19)	5.70 (5)	4.80 (2)	12.50 (5)	5.30 (2)	
Other	3.40 (25)	2.50 (7)	3.20 (8)	1.10 (1)	0.00	0.00	0.00	
**Level of Education**[3]								0.12
Postgrad	18.30 (134)	17.40 (48)	19.80 (49)	18.40 (16)	21.40 (9)	5.00 (2)	26.30 (10)	
Undergrad	32.10 (235)	30.10 (83)	36.30 (90)	32.20 (28)	31.00 (13)	12.50 (5)	42.10 (16)	
A level/NVQ	21.30 (156)	25.70 (71)	19.40 (48)	24.10 (21)	9.50 (4)	25.00 (10)	5.30 (2)	
Diploma/BTEC	10.70 (78)	9.80 (27)	8.90 (22)	8.00 (7)	16.70 (7)	32.50 (13)	5.30 (2)	
O level/GCSE	11.10 (81)	10.90 (30)	9.70 (24)	12.60 (11)	9.50 (4)	17.50 (7)	13.20 (5)	
No formal	2.10 (15)	1.40 (4)	2.00 (5)	3.40 (3)	2.40 (1)	5.00 (2)	0.00	
**Service type**								0.17
Outpatient	91.00 (665)	82.60 (228)	96.80 (240)	97.70(85)	100.00(42)	92.50 (37)	86.80 (33)	
Inpatient	6.00 (44)	13.40 (37)	0.40 (1)	1.10 (1)	0.00	5.00 (2)	7.90 (3)	
Day	2.20 (16)	2.50 (7)	2.80 (7)	1.10 (1)	0.00	2.50 (1)	0.00	
ETT	0.80 (6)	1.40 (4)	0.00	0.00	0.00	0.00	5.30 (2)	
**Weight trend**								0.23
Increasing	26.50 (194)	17.00 (47)	37.90 (94)	12.60 (11)	76.20 (32)	5.00 (2)	21.1 (8)	
Unchanging	38.00 (279)	42.40 (117)	32.70 (82)	42.50 (37)	11.90 (5)	55.00 (22)	42.1 (16)	
Decreasing	28.70 (210)	33.00 (91)	23.00 (57)	41.50 (36)	4.80 (2)	35.00 (14)	26.3 (10)	
**Alcohol consumption**								0.11
Never	27.80 (203)	28.30 (78)	23.40 (58)	29.90 (26)	23.80 (10)	55.00 (22)	23.7 (9)	
Monthly/less	27.80 (203)	27.20 (75)	25.80 (64)	36.80 (32)	33.30 (14)	27.50 (11)	18.4 (7)	
x2-4/month	22.60 (165)	20.70 (57)	26.20 (65)	19.50 (17)	31.10 (13)	15.00 (6)	18.4 (7)	
x2-3/week	14.20 (104)	15.20 (42)	16.90 (41)	11.50 (10)	4.80 (2)	0.00	21.1 (8)	
x4+/week	2.50 (18)	2.90 (8)	2.80 (7)	1.10 (1)	0.00	0.00	5.30 (2)	

AN-R=Anorexia Nervosa-Restrictive Subtype, BN=Bulimia Nervosa, A-AN=Atypical Anorexia Nervosa, BED=Binge Eating Disorder, ARFID=Avoidant Restrictive Food Intake Disorder, AN-BP=Anorexia Binge-Purge Subtype, *V*= Cramer' V. [1]*n*=1 prefer not to say; [2]*n*=70 N/A or unsure or prefer not to say; [3]*n*=4 N/A.

Table S3. Family history of Autism, Support needs (verbal and visual), absence from work or school due to ED, history of one or more suicide attempt, and for all cases, shown overall and separately for the six groups. Cells where *n*<5 are orange.

	**Overall, % (n)**	**AN-R, % (n)**	**BN, % (n)**	**A-AN, % (n)**	**BED, % (n)**	**ARFID, % (n)**	**AN-BP, % (n)**	**Effect size, *v***
Family history of Autism	24.10 (176)	22.80 (63)	21.40 (53)	29.90 (26)	31.00 (13)	32.50 (13)	21.1 (8)	0.08
Verbal support needs	3.10 (23)	2.60 (7)	2.90 (7)	2.20 (2)	7.20 (3)	5.00 (2)	10.60 (4)	0.16
Visual support needs	2.60 (19)	2.50 (7)	2.50 (6)	3.30 (3)	7.20 (3)	5.00 (2)	0.00	0.12
Absence from work[1]	26.00 (190)	31.20 (86)	21.80 (54)	27.60 (24)	11.90 (5)	20.00 (8)	34.20 (13)	0.12
Absence from school	18.60 (136)	25.70 (71)	12.50 (31)	14.90 (13)	16.70 (7)	17.50 (7)	18.40 (7)	0.16
Suicide attempt	25.30 (185)	21.40 (59)	27.40 (68)	25.30 (22)	28.60 (12)	22.50 (9)	39.50 (15)	0.06
Smoking	12.90 (94)	9.80 (27)	16.10 (40)	14.90 (13)	4.80 (2)	17.50 (7)	13.20 (5)	0.11

V= Cramer' V, [1]*n*=126 not applicable.

Table S4. Unadjusted and adjusted linear regression models for the EDE-Q in the AN-R sample.

AN-R SAMPLE	*(n)*	*B*	*(95% BCa CI)*	*Adjusted R^2*	*ΔR^2*	*P*
Model 1 (Unadjusted)	(254)	.160	(.088, .240)	.063	.067	< .001
Model 2 (+ age)	(254)	.149	(.077, .230)	.082	.023	< .001
Model 3 (+ gender)	(254)	.147	(.075, .230)	.094	.015	< .001
Model 4 (+ ED duration)	(254)	.122	(.045, .205)	.137	.046	< .001

Table S5. Unadjusted and adjusted linear regression models for the EDE-Q in the BN sample.

BN SAMPLE	*(n)*	*B*	*(95% BCa CI)*	*Adjusted R^2*	*ΔR^2*	*P*
Model 1 (Unadjusted)	(232)	.065	(.008, .121)	.018	.022	.032
Model 2 (+ age)	(232)	.066	(.009, .120)	.015	.001	.031
Model 3 (+ gender)	(232)	.067	(.029, .122)	.011	.000	.029
Model 4 (+ ED duration)	(232)	.055	(-.007, .115)	.033	.026	.074

Table S6. Unadjusted and adjusted linear regression models for the EDE-Q in the full sample.

FULL SAMPLE	*(n)*	*B*	*(95% BCa CI)*	*Adjusted R^2*	*ΔR^2*	*P*
Model 1 (Unadjusted)	(680)	.088	(.043, .133)	.019	.020	< .001
Model 2 (+ age)	(680)	.087	(.042, .133)	.018	.000	< .001
Model 3 (+ gender)	(680)	.090	(.044, .136)	.022	.006	< .001
Model 4 (+ ED duration)	(680)	.082	(.036, .129)	.032	.012	< .001
Model 5 (+ Diagnosis)	(680)	.097	(.057, .137)	.234	.206	< .001

Table S7. Unadjusted and adjusted linear regression models for the CORE-10 in the AN-R sample.

AN-R SAMPLE	*(n)*	*B*	*(95% BCa CI)*	*Adjusted* R^2	ΔR^2	*P*
Model 1 (Unadjusted)	(254)	.909	(.633, 1.195)	.133	.137	< .001
Model 2 (+ age)	(254)	.884	(.600, 1.157)	.137	.007	< .001
Model 3 (+ gender)	(254)	.881	(.592, 1.168)	.137	.004	< .001
Model 4 (+ ED duration)	(254)	.783	(.487, 1.070)	.179	.045	< .001

Table S8. Unadjusted and adjusted linear regression models for the CORE-10 in the BN sample.

BN SAMPLE	*(n)*	*B*	*(95% BCa CI)*	*Adjusted* R^2	ΔR^2	*P*
Model 1 (Unadjusted)	(232)	.682	(.399, .972)	.064	.068	< .001
Model 2 (+ age)	(232)	.690	(.401, 1.003)	.063	.003	< .001
Model 3 (+ gender)	(232)	.702	(.401, 1.026)	.063	.004	< .001
Model 4 (+ ED duration)	(232)	.683	(.394, .975)	.062	.002	< .001

Table S9. Unadjusted and adjusted linear regression models for the CORE-10 in the full sample.

FULL SAMPLE	*(n)*	*B*	*(95% BCa CI)*	*Adjusted* R^2	ΔR^2	*P*
Model 1 (Unadjusted)	(680)	.755	(.595, .914)	.085	.086	< .001
Model 2 (+ age)	(680)	.750	(.589, .912)	.086	.002	< .001
Model 3 (+ gender)	(680)	.761	(.598, .924)	.091	.007	< .001
Model 4 (+ ED duration)	(680)	.728	(.571, .884)	.103	.013	< .001
Model 5 (+ Diagnosis)	(680)	.744	(.590, .900)	.119	.022	< .001

Table S10. Unadjusted and adjusted linear regression models for the WSAS in the AN-R sample.

AN-R SAMPLE	*(n)*	*B*	*(95% BCa CI)*	*Adjusted R^2*	*ΔR^2*	*P*
Model 1 (Unadjusted)	(254)	1.352	(.940, 1.763)	.119	.122	< .001
Model 2 (+ age)	(254)	1.316	(.916, 1.693)	.122	.006	< .001
Model 3 (+ gender)	(254)	1.309	(.908, 1.697)	.126	.007	< .001
Model 4 (+ ED duration)	(254)	1.117	(.700, 1.502)	.192	.069	< .001

Table S11. Unadjusted and adjusted linear regression models for the WSAS in the BN sample.

BN SAMPLE	*(n)*	*B*	*(95% BCa CI)*	*Adjusted R^2*	*ΔR^2*	*P*
Model 1 (Unadjusted)	(232)	1.202	(.748, 1.717)	.097	.101	< .001
Model 2 (+ age)	(232)	1.184	(.742, 1.675)	.101	.008	< .001
Model 3 (+ gender)	(232)	1.207	(.771, 1.722)	.104	.007	< .001
Model 4 (+ ED duration)	(232)	1.118	(.666, 1.645)	.123	.022	< .001

Table S12. Unadjusted and adjusted linear regression models for the WSAS in the full sample.

FULL SAMPLE	*(n)*	*B*	*(95% BCa CI)*	*Adjusted R^2*	*ΔR^2*	*P*
Model 1 (Unadjusted)	(680)	1.272	(.972, 1.543)	.101	.103	< .001
Model 2 (+ age)	(680)	1.279	(.979, 1.544)	.102	.002	< .001
Model 3 (+ gender)	(680)	1.287	(.986, 1.550)	.103	.002	< .001
Model 4 (+ ED duration)	(680)	1.215	(.928, 1.475)	.128	.027	< .001
Model 5 (+ Diagnosis)	(680)	1.223	(.952, 1.470)	.142	.021	< .001

Table S13. Multicollinearity diagnostics for the final regression models.

Sample	Outcome	Predictor	Tolerance	VIF
**AN-R Sample**	EDE-Q	AQ-10	.952	1.050
		Age	.421	2.376
		Gender	.966	1.036
		ED duration	.429	2.334
	CORE-10	AQ-10	.952	1.050
		Age	.421	2.376
		Gender	.966	1.036
		ED duration	.429	2.334
	WSAS	AQ-10	.952	1.050
		Age	.421	2.376
		Gender	.966	1.036
		ED duration	.429	2.334
**BN Sample**	EDE-Q	AQ-10	.968	1.033
		Age	.437	2.289
		Gender	.993	1.007
		ED duration	.430	2.327
	CORE-10	AQ-10	.968	1.033
		Age	.437	2.289
		Gender	.993	1.007
		ED duration	.430	2.327
	WSAS	AQ-10	.968	1.033
		Age	.437	2.289
		Gender	.993	1.007
		ED duration	.430	2.327
**Full sample**	EDE-Q	AQ-10	.978	1.022
		Age	.464	2.156
		Gender	.965	1.037
		ED duration	.471	2.123
		AN-BP	.916	1.092
		BN	.777	1.286
		A-AN	.855	1.170
		BED	.897	1.114
		ARFID	.895	1.117
	CORE-10	AQ-10	.978	1.022
		Age	.464	2.156
		Gender	.965	1.037
		ED duration	.471	2.123
		AN-BP	.916	1.092
		BN	.777	1.286
		A-AN	.855	1.170
		BED	.897	1.114
		ARFID	.895	1.117
	WSAS	AQ-10	.978	1.022
		Age	.464	2.156
		Gender	.964	1.037
		ED duration	.471	2.123
		AN-BP	.916	1.092

	BN	.778	1.285
	A-AN	.855	1.170
	BED	.897	1.114
	ARFID	.897	1.115

Note. Tolerance and VIF values are taken from the normal (non-bootstrap) regression output for the final model. For AN-R and BN models, values are from Model 4; for full-sample models, values are from Model 5. Diagnosis categories are shown using the labels entered in the regression model.

Supplementary Materials S1. Replication of Tchanturia et al. [6]

This appendix reports a sensitivity analysis replicating the analytical approach used by Tchanturia et al. [6] to examine the associations between autistic traits and clinical outcomes. While the main analyses used multiple linear regression models to control for age, gender, and ED duration, this additional analysis applied a direct correlational approach to enable comparison with previous findings in a similar clinical population.

Kendall's tau-b (τ_b) correlation co-efficients were used to examine associations between autistic traits (AQ-10) and measures of ED psychopathology (EDE-Q), psychological distress (CORE-10), and functional impairment (WSAS) across diagnostic groups (AN-R, BN, and full sample). This approach was selected to align with the original analytical strategy used by Tchanturia et al. [6]. Given multiple comparisons, statistical significance was interpreted with reference to a Bonferroni-adjusted threshold.

Across all diagnostic groups, higher AQ-10 scores were significantly and positively associated with greater eating disorder psychopathology, psychological distress, and functional impairment. Associations were generally strongest for psychological distress and functional difficulties. In the full sample, correlations were small but consistent across all outcomes, with larger coefficients observed in the diagnostic subgroups (AN-R and BN). A visual summary of these associations is presented in Figure S1, and full results are reported in Table S14.

Overall, findings from this sensitivity analysis were consistent with the main regression models, supporting the robustness of the association between autistic traits and clinical measures across sample groups.

Figure S1. Visual synthesis of the Kendall's tau-b correlation analysis findings. Arrow size indicates the strength of correlation. Correlations are based on the AQ-10 scores withing each of the diagnostic samples.

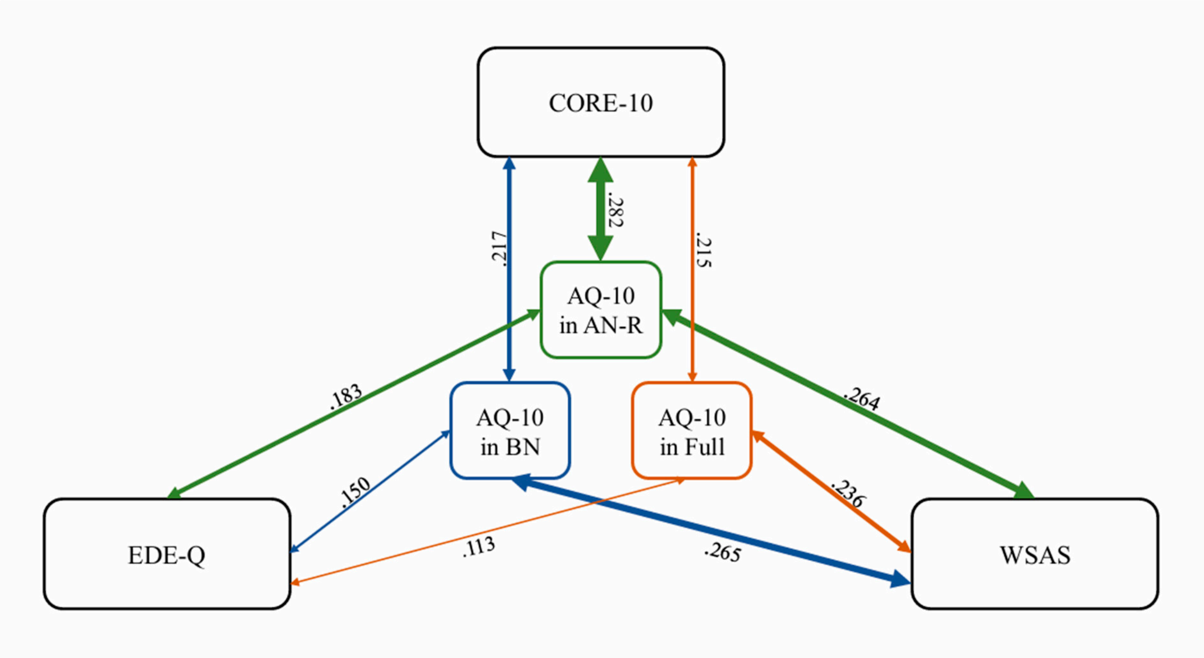

Table S14. Correlation between AQ-10 scores and psychopathology across diagnoses.

Measure	Statistic	AN-R	BN	Full Sample
EDE-Q	**Kendall's (τ_β)**	.183	.150	.113
	**p-value**	<.001	<.001	<.001
CORE10	**Kendall's (τ_β)**	.282	.217	.215
	**p-value**	<.001	<.001	<.001
WSAS	**Kendall's (τ_β)**	.264	.265	.236
	**p-value**	<.001	<.001	<.001

Note. Kendall's tau-b correlations are reported. A Bonferroni correction was applied to account for multiple comparisons across nine tests, resulting in an adjusted significance threshold of α = .006 (.05/9). All reported correlations remained statistically significant following correction. *p*-values are two-tailed.

Supplementary Materials S2

Patient Intake Form, excluding CORE10, EDE-Q, AQ-10, and WSAS.

1. Trust ID (please enter in the format xx-xx-xx) *
2. Which service are you currently using*
    - SLaM Eating Disorders Outpatients Service
    - SLaM Eating Disorders Inpatient Service (Bethlem Royal Hospital)
    - SLaM Eating Disorders Day Services
    - SLaM Eating Disorders Enhanced Treatment Team (ETT)
3. Clinician Name (if known)
4. Timepoint
    - Assessment
    - Treatment Start
    - Mid Treatment
    - Treatment End
    - Follow Up 1
    - Follow Up 2
    - Other: ___________
5. Date of Birth*
6. Age*
7. Gender*
    - Female
    - Male
    - Non-binary
    - Other: ___________
8. Pro-noun*
    - She/Her
    - He/Him
    - They/Them
    - Other: ___________
9. Sexual orientation*
    - Heterosexual
    - Gay
    - Lesbian
    - Bisexual
    - Asexual
    - Unsure or prefer not to say
    - Other: ___________
10. Ethnicity*
    - White British
    - White Irish
    - White Other
    - Black Afro-Caribbean
    - Black African
    - Black British
    - Mixed
    - Asian
    - Other: ___________
11. Is English your first language? *
    - Yes
    - No
12. What is your marital status? *
    - Married
    - Living together
    - Single
    - Divorced
    - Separated

- o Widowed
- o Other: ___________

13. What is your current employment status? *
    - o Full time
    - o Part time
    - o Unemployed
    - o Student
    - o Retired
    - o Sick leave
    - o Housewife/husband
    - o Other: ___________
14. What is the highest level of education you completed? *
    - o No qualifications
    - o O Level / GCSE
    - o A Level / NVQ
    - o Diploma / BTEC
    - o University Degree
    - o Postgraduate Degree
    - o Other: ___________
15. How many years of education have you received?
    (Include primary school, secondary school and any further education you have had.)
16. Have you had to take time off from <u>school</u> due to your eating disorder? *
    - o Yes
    - o No
17. Have you had to take time off from <u>work</u> due to your eating disorder? *
    - o Yes
    - o No
    - o Not applicable
18. Who lives in your household with you? *
    - o Parent(s)
    - o Sibling(s)
    - o Housemate(s)/friend(s)
    - o Spouse
    - o Partner
    - o Parent(s) and sibling(s)
    - o Parent(s), sibling(s), grandparent(s)
    - o Live alone
    - o Child/children
    - o Spouse and child/children
    - o Other: ___________
19. Do you own, rent or share your accommodation? *
    - o Own
    - o Rent
    - o Share/live with family (e.g., with parents)
    - o Council accommodation
    - o Sheltered or temporary accommodation
    - o Other: ___________
20. Has anyone in your family been diagnosed with Autism? *
    - o Yes
    - o No
21. Do you have any children? *
    - o Yes
    - o No
22. For how many years have you had an eating disorder? *
23. Have you had any professional help for eating disorders before? (choose one or more)
    - o Hospital admission(s)
    - o Day patient treatment

- Outpatient treatment
- No
- Other: ___________

24. Have you ever attempted suicide? *
    - Yes
    - No
25. Accessible information needs: Please identify if you need support in **verbal** communication *
    - No accessible support needs
    - Interpreter needed
    - Hearing loss or hearing aids used
    - Contact via text or email only
    - Other: ___________
26. Accessible information needs: Please identify if you need support in **visual** communication: *
    - No accessible support needs
    - Large font size
    - Contact via text or email only
    - Other: ___________
27. Please specify any other support needs
28. Do you currently have any mental health diagnoses other than your eating disorder? If yes, please give details below
29. Current medications and doses (if none, leave blank)
30. Current weight (in kg), approximate is fine
31. Current height (in cm), approximate is fine
32. Recently has your weight been
    - Increasing
    - Decreasing
    - Staying about the same
33. Lowest weight you have ever been? (in kg) Approximate is fine
34. What was your height at the time? (in cm)
35. Highest weight you have ever been? (in kg) Approximate is fine
36. What was your height at the time? (in cm)
37. Do you currently smoke tobacco?
    - Yes
    - No
